# Population Preference of Surgeon's Gender for Surgical Care and Their Attitudes Toward Female Surgeons in Taif, Saudi Arabia

**DOI:** 10.7759/cureus.28017

**Published:** 2022-08-14

**Authors:** Layla M Alkhaldi, Abeer I Alsulaimani, Wahaj A Altalhi, Ghaida M Alghamdi, Noura N Alqurashi, Tamer M Abdelrahman

**Affiliations:** 1 Medicine, Taif University, Taif, SAU; 2 Surgery, Benha Teaching Hospital, Benha, EGY; 3 Surgery, College of Medicine, Taif University, Taif, SAU

**Keywords:** obstetrics, breast cancer, patient preferences, gender bias, surgery

## Abstract

Introduction

One contributing factor that has led to a reduction in the number of females entering the profession of surgery is discrimination against female surgeons. Little is known about the practices, attitudes, and perceptions of the Saudi population toward gender discrimination in the field of surgery. The aim of this study was to assess the practices, attitudes, and perceptions of the Taif population in choosing a surgeon based on their gender.

Materials and methods

An online pretested questionnaire was randomly sent to the participants living in Taif city. Collected data were subjected to scrutiny to check participants' study inclusion criteria. All the data were subjected to statistical analysis by an independent biostatistician. Pearson's chi-square test was used to search for a statistically significant association between categorical variables.

Results

About 49.5% of the participants preferred a surgeon of the same gender when consulting for a non-emergency visit to a surgery clinic, whereas females significantly preferred a female surgeon (p < 0.001). The most common reason to choose surgeons of the same gender was comfort, followed by ease of talking. About 71.8% of the participants preferred female surgeons for ''sensitive'' surgical cases such as genital, obstetric, or sexual disorders, whereas 12.1% preferred male surgeons.

Conclusion

Females have shown progress in the field of surgery, but there is still much to be done to convert the surgical workplace to be more supportive of women so that they contribute their best effort.

## Introduction

Males have primarily dominated the medical field, and this long-standing unequal gender order has resulted in institutional impediments that prevent women from progressing in the medical field [1]. Reports show that about 67% of female surgeons still face discrimination, despite the fact that more women are graduating from medical school, more women are represented in leadership roles, and more people are aware of gender bias in surgery [2-4]. In the surgical profession, implicit gender bias is very prevalent [5]. In addition to describing how men and women should act, gender stereotypes prescribe specific behaviors. Men are prescriptive for assertiveness, competitiveness, and independence, while women are prescriptive for warmth, kindness, and support [6,7]. This is likely due to the fact that agency is required for leadership and career success, while communality is essential for caring for others' well-being [8,9].

Discrimination against female surgeons has been a contributing factor to a decrease in the number of females entering the field of surgery [10]. As a result, there is an under-representation of females in surgery, which compromises quality mentorship and creates a hostile situation that further instills barriers to entry [11]. Attrition among female surgical residents is around 25% greater than among male residents because of gender bias [12]. Shortage of surgeons leads to an increase in burnout and medical errors [13]. Dropout rates for female surgeons are also alarming, as these surgeons have valuable features such as higher surgical outcomes due to greater physician-patient communication and more patient-centered care [14,15]. Moreover, diversification can further fulfill the demands of a diverse patient community, as some female patients actively prefer female doctors as their surgeons [15].

Several studies have examined the differences between the work habits of male and female doctors. Patient preference for the genders of various health providers was studied in a Dutch study in 1997. The study reported that patients who favored female doctors did so not just because they felt more comfortable having their sensitive health information examined by a woman doctor, and also found that female doctors tend to be friendlier and more approachable [16]. Even after more than 25 years, the same communication techniques of doctors are still being studied [17]. There is an even greater disparity between expected feminine behavior and the attributes that embody the typical surgeon for females in the medical field. Evidence shows that surgeons have been found to be tougher, more stubborn, and less empathic than non-surgeons [18]. Male ideals are modeled for women as standards for success or skill in fields where males predominate [19,20]. A study done by Hoffman et al. in the USA reported that up to 17% of patients who need colorectal surgery choose their surgeon based on the gender of the surgeon. Female patients were 1.47 times more likely than male patients to request a surgeon based on gender [21]. According to Groutz et al., one-third of female patients who attended breast clinics in two university-affiliated tertiary hospitals choose a female breast surgeon for their breast assessment. The prime reason behind the preference for examinees of the same gender was embarrassment. However, it was reported that preference for a female surgeon is less prominent for breast surgeries, where the surgeon's professional qualifications were the predominant consideration than the gender [22]. Shared decision-making, as demonstrated by the high level of patient satisfaction with such approaches, is clearly a helpful tool for healthcare professionals. Health disparities may be worsened by some preferences, which may have an impact on the usage of healthcare services [23]. Evidence shows that depending on the gender of the healthcare professional, patients may opt out of required procedures such as a colonoscopy [15]. It is plausible that some patients would not seek medical attention for a critical issue such as breast examination or mammography.

Barriers to the advancement of female surgeons persist despite the serious implications for the general well-being framework. To provide optimal patient-centered treatment, greater research into the factors that contribute to the gender differences in how some patients respond to sensitive physical examinations is warranted. Gender bias in surgery can be better understood by examining the preferences of patients rather than just relying on one perspective. Thus, this study aimed to assess gender preferences when selecting surgeons for consultation and surgeries related to breast cancer and hemorrhoids among the population of Taif, Saudi Arabia.

## Materials and methods

A population-based, cross-sectional study was conducted among participants from Taif city, Saudi Arabia. Permission for conducting the study was taken from the Research and Ethics Committee of Taif University. The study population included adults aged 18 years and above living in Taif city. A convenience sampling was used to record the responses based on the above eligibility criteria. An online pretested self-reported anonymous questionnaire was used to collect responses from the participants to assess participants' preferences in choosing their surgeon. The data collection was done between June 2022 and July 2022.

The questionnaire consisted of five parts. The first part included the demographic details of the participants (age, gender, marital status, city, nationality, occupation, and educational level). The second part included items that measured participants’ attitudes in choosing surgeons of the same gender. The third part consisted of questions related to participants’ attitudes regarding the gender of surgeon preference in different surgical situations. The fourth part had five-point Likert scale items that measured the attitude of the population toward female surgeons. The final part included participants' practices related to choosing surgeons. The questionnaire was pretested on 15 participants, which showed good reliability (Cronbach's alpha = 0.854).

Statistical analysis and data management

All the collected information was tabulated on a Microsoft Excel sheet (Microsoft Corporation, Redmond, WA) and then transferred to IBM Statistical Package for the Social Sciences version 23 (IBM Corp., Armonk, NY) for data analysis. Descriptive statistics in the form of frequencies and percentages using suitable tables and figures were used to represent categorical data. Continuous variables were presented using mean and standard deviation. Comparison of continuous variables between categorical variables was evaluated using the Student's test and/or analysis of variance. Any possible association between categorical variables was measured using Pearson's chi-square test. A p-value < 0.05 was considered statistically significant.

## Results

Our analysis included responses from 635 participants, in which the sociodemographic analysis showed that 340 (53.5%) belonged to the age group of 18-28 years, 546 (86%) were females, 340 (53.5%) were single, 464 (73.1%) had university level educational qualifications, and 202 (31.8%) were employed (Table 1).

**Table 1:** Sociodemographic details of the participants

		N	%
Age	18-28	340	53.5
	29-39	125	19.7
	40-50	105	16.5
	>50	65	10.2
Gender	Female	546	86.0
	Male	89	14.0
Marital status	Single	340	53.5
	Married	267	42.0
	Divorced	19	3.0
	Widowed	9	1.4
Educational level	General education (primary, intermediate, secondary)	131	20.6
	University	464	73.1
	Postgraduate	40	6.3
Employment status	Employed	202	31.8
	Student	234	36.9
	Unemployed	199	31.3

The analysis showed that 317 (49.9%) participants had consulted or undergone treatment by a surgeon. When asked about the body regions that they preferred to be examined by the surgeon of the same gender, the most commonly reported region was genitals (61.1%), followed by the pelvic region (51.5%), chest region (46.0%), and abdomen (33.7%). The region that had given the least preference was the extremities (3.3%), whereas about 16.4% did not have any gender preferences for surgery (Table 2).

**Table 2:** Surgery-related history

Surgery-related history		N	%
Have consulted or undergone treatment by a surgeon or surgeons		317	49.9
Body region that would be examined by a surgeon of the same sex	Face region	126	19.8
	Mouth	156	24.6
	Head region	112	17.6
	Neck region	99	15.6
	Chest region	292	46.0
	Abdomen	214	33.7
	Pelvic region	327	51.5
	Genitals	388	61.1
	Extremity	21	3.3
	Back	151	23.8
	I did not have any preferences	104	16.4
Reasons for preferring gender of the same gender (n = 531)	Comfortable	379	71.4
	Modesty	315	59.3
	I can talk openly with a surgeon of the same gender	316	59.5
	Do not believe that a surgeon of the same sex has a greater understanding of the symptoms	155	29.2
	Confusion	101	19.0

When we assessed the type of doctor preferred for regular non-emergency visits to the surgery clinic, it was found that female participants preferred female surgeons (96.8%) significantly higher than males who preferred male surgeons (45.4%) (p < 0.001). It was found that 102 (55.4%) participants preferred female surgeons for emergency surgical cases, and the majority (94.1%) were female participants, which showed a statistically significant relationship (p < 0.001). When asked about the surgeon they preferred for "sensitive" surgical cases (genital, obstetric, or sexual problems), about 71.8% mentioned female surgeons, whereas 12.1% needed a male surgeon. Female participants significantly preferred female surgeons for this purpose (97.6%), and males preferred male surgeons (71.4%) compared to surgeons of the opposite gender (p < 0.001). In the event of a minor surgical procedure (such as draining an abscess), the majority of female participants preferred a female surgeon to do the surgery (95.7%), whereas male participants did not have much preference for the same gender (36.1%) (p < 0.001). The same gender preferences were also noted for major surgery (surgical intervention or laparoscopic surgery) among the participants, where female participants significantly preferred female surgeons (97.2%) compared to males who preferred male surgeons (29.4%) (p < 0.001; Table 3).

**Table 3:** Participants' gender preferences for surgery

		Gender of participant		Total	P-value
		Female	Male		
Surgeon preferred for regular non-emergency visits to the surgery clinic	Female	210 (96.8%)	7 (3.2%)	217 (34.2%)	<0.001
	Male	53 (54.6%)	44 (45.4%)	97 (15.3%)	
	No preferences	283 (88.2%)	38 (11.8%)	321 (50.6%)	
Surgeon preferred for emergency surgical cases	Female	96 (94.1%)	6 (5.9%)	102 (55.4%)	<0.001
	Male	132 (72.9%)	49 (27.1%)	181 (28.5%)	
	No preferences	318 (90.3%)	34 (9.7%)	353 (55.4%)	
Surgeon preferred for "sensitive" surgical cases (genital, obstetric, or sexual problems)	Female	445 (97.6%)	11 (2.4%)	456 (71.8%)	<0.001
	Male	22 (28.6%)	55 (71.4%)	77 (12.1%)	
	No preferences	79 (77.5%)	23 (22.5%)	102 (16.1%)	
Surgeon preferred in the event that you need a minor surgical procedure (such as draining an abscess)	Female	178 (95.7%)	8 (4.3%)	186 (29.3%)	<0.001
	Male	69 (63.9%)	39 (36.1%)	108 (17%)	
	No preferences	299 (87.7%)	42 (12.3%)	341 (53.7%)	
Surgeon preferred for major surgery (surgical intervention or laparoscopic surgery)	Female	138 (97.2%)	4 (2.8%)	142 (22.4%)	<0.001
	Male	120 (70.6%)	50 (29.4%)	170 (26.8%)	
	No preferences	288 (89.2%)	35 (10.8%)	323 (50.9%)	

We used a five-point Likert scale to assess the participants' perceptions of female surgeons' competence and other experiences in the workplace (Table 4). About 45% of the participants showed disagreement with the statement “Female surgeons are less qualified and should make more effort than male surgeons to prove their qualifications as physicians.” At the same time, 34.1% showed agreement with the same. It was agreed by 48.5% that female surgeons and surgical trainees find it harder to command authority as patients and hospital staff tended to dismiss them and trust more in male colleagues, whereas only 25.1% disagreed with this. About 42.4% did not agree that female surgeons need to work harder than male surgeons to establish their legitimacy as doctors. However, more than half of the participants (58.6%) had the opinion that female surgeons are detail-oriented, empathetic, and more nurturing toward patients, bringing important skills to surgery that differed from male peers. Similarly, we observed that 62.9% of the participants had the view that female surgeons are preferred and actively chosen by some patients for reasons including more delicate surgical work or being better equipped to understand and look after pregnant patients. About 45.1% of the participants believed that female surgeons and trainees get lack of support in their workplace.

**Table 4:** Participants’ perceptions of females surgeons’ competence and other experiences in the workplace

	Strongly agree	Agree	Neutral	Disagree	Strongly disagree	Mean (SD)
Female surgeons are less qualified and should make more effort than male surgeons to prove their qualifications as physicians	105 (16.5%)	112 (17.6%)	132 (20.8%)	138 (21.7%)	148 (23.3%)	2.82 (1.40)
Female surgeons and surgical trainees find it harder to command authority as patients and hospital staff tended to dismiss them and trusted more in male colleagues	99 (15.6%)	209 (32.9%)	168 (26.5%)	102 (16.1%)	57 (9.0%)	3.30 (1.18)
Female surgeons need to work harder than male surgeons to establish their legitimacy as doctors	76 (12.0%)	113 (17.8%)	177 (27.9%)	139 (21.9%)	130 (20.5%)	2.79 (1.29)
Female surgeons are detail-oriented, empathetic, and more nurturing toward patients, bringing important skills to surgery that differed from male peers	169 (26.6%)	203 (32.0%)	183 (28.8%)	51 (8.0%)	29 (4.6%)	3.68 (1.09)
Female surgeons are preferred and actively chosen by some patients for reasons including more delicate surgical work or being better equipped to understand and look after pregnant patients	196 (30.9%)	203 (32.0%)	162 (25.5%)	45 (7.1%)	29 (4.6%)	3.78 (1.10)
Female surgeons and trainees get lack of support in their workplace	102 (16.1%)	184 (29.0%)	226 (35.6%)	74 (11.7%)	49 (7.7%)	3.34 (1.12)

We assessed participants' preferences for surgeons' gender for surgeries related to breast cancer, and it was found that 36.2% preferred female surgeons compared to male surgeons (14.2%). But this did not show any statistically significant association with participants' gender (p = 0.890). For preferences for surgeons' gender for surgeries related to hemorrhoid surgery, 51.5% mentioned female surgeons are the best, and among this, 98.8% were female participants (p < 0.001; Table 5).

**Table 5:** Preferences for breast cancer and hemorrhoid surgery and its relationship with participants’ gender

			Gender		Total	P-value
			Female	Male		
Surgeon's gender preferences for breast cancer surgery	No	N	271	44	315	0.890
		%	86.0%	14.0%	49.6%	
	Yes, female surgeons are always the best	N	199	31	230	
		%	86.5%	13.5%	36.2%	
	Yes, male surgeons are always the best	N	76	14	90	
		%	84.4%	15.6%	14.2%	
Surgeon's gender preferences for hemorrhoid surgery	No	N	177	34	211	<0.001
		%	83.9%	16.1%	33.2%	
	Yes, female surgeons are always the best	N	323	4	327	
		%	98.8%	1.2%	51.5%	
	Yes, male surgeons are always the best	N	46	51	97	
		%	47.4%	52.6%	15.3%	

The most cited reason for surgeons' gender preferences for breast cancer surgery was that they "can talk openly with a surgeon of the same gender" (51.2%), whereas 95.1% of the participants believed that female surgeons are always the best in this regard. The next reason that was cited more commonly was "confident" (40.9%), in which 71.0% of the participants believed that female surgeons are always the best in this regard. About 103 participants cited "competent" as a reason for choosing a surgeon, where 50.5% of them believed that male surgeons are always the best in this regard. Superior professional skills were cited by 66 (20.6%) participants, and 56.1% believed that male surgeons are always the best. Among those who cited the reason as "examine patients better" (26.6%), about 62.4% believed that female surgeons are always the best. The most cited reason for surgeons' gender preferences for hemorrhoid surgery was that they "can talk openly with a surgeon of the same gender" (65.8%), followed by "confident" (33.5%) and competent (21.2%). The least cited reason was "bad experience with surgeons of opposite gender in the past" (5.2%) (Table 6).

**Table 6:** Reasons for choosing surgeon’s gender for breast cancer and hemorrhoid surgery

Reasons for choosing a surgeon	Surgeon's gender preferences for breast cancer surgery			Surgeon's gender preferences for hemorrhoid surgery		
	Yes, female surgeons are always the best	Yes, male surgeons are always the best	Total	Yes, female surgeons are always the best	Yes, male surgeons are always the best	Total
Fearless	50	42	92	47	39	86
	54.3%	45.7%	28.7%	54.7%	45.3%	20.3%
Confident	93	38	131	99	43	142
	71.0%	29.0%	40.9%	69.7%	30.3%	33.5%
Higher level of patience	51	31	82	50	25	75
	62.2%	37.8%	25.6%	66.7%	33.3%	17.7%
More experienced	39	43	82	33	36	69
	47.6%	52.4%	25.6%	47.8%	52.2%	16.3%
Competent	51	52	103	45	45	90
	49.5%	50.5%	32.2%	50.0%	50.0%	21.2%
Can talk openly with a surgeon of the same gender	156	8	164	247	32	279
	95.1%	4.9%	51.2%	88.5%	11.5%	65.8%
Bad experience with surgeons of opposite gender in the past	12	10	22	14	8	22
	54.5%	45.5%	6.9%	63.6%	36.4%	5.2%
Superior professional skills	29	37	66	30	34	64
	43.9%	56.1%	20.6%	46.9%	53.1%	15.1%
Examines patients better	53	32	85	57	24	81
	62.4%	37.6%	26.6%	70.4%	29.6%	19.1%

## Discussion

The primary objective of our research was to get a more in-depth understanding of patient perspectives to provide female surgeons with guidance on how to engage with patients in the most effective manner. The findings of our study show that the majority of the female participants significantly preferred female surgeons, whereas males did not show many such preferences. Evidence shows that although women physicians spend more time with their patients and typically use a patient-centered approach, they are not rated as highly as their male counterparts [24,25]. At the same time, other studies indicate that the gender of the physician has no impact on patient satisfaction [26]. There is some evidence that the doctor-patient relationship and patient satisfaction may be influenced by the doctor-patient communication styles [27]. In our study, the majority of the participants preferred a surgeon of the same gender to examine sensitive body regions such as genitals and pelvic region, whereas there were no such major preferences observed for areas such as extremities, mouth, head, and neck regions. Studies show that different communication styles are used by surgeons during surgical procedures, with female surgeons delivering more patient-centered care than male surgeons [24,28]. The most commonly cited reason for preferring surgeons of the same gender was that they talked openly with surgeons of the same gender during physical examination. This finding is consistent with a Dutch study that supports "There are virtually no sex preferences for the more “instrumental” health professions (e.g. surgeons and anesthetists)," which also reported the same reason while consulting a primary care physician [16].

Due to a lack of research on this topic and contradictory findings in the existing literature, the relationship between patient gender, surgeon gender, and perceived quality of care is poorly understood. There is evidence that female-female doctor-patient pairs received higher satisfaction ratings than male-male doctor pairs in an aftercare study of cancer patients who had been randomly allocated to a male or female physician [29]. Our findings are consistent with most previous studies reporting strong female patient-female physician preferences [30-33] but in contrast to the other two studies that reported male patients to have a stronger same-gender physician preference than females [16,34]. In a qualitative study, females said they wanted a female physician as they believed that a female physician would pay more attention to their concerns and have a solid insight into their problems [30]. In our survey, we chose to study breast cancer and hemorrhoid surgery to see the influence of patient preferences on the surgeon's gender. Although breast cancer is very rare among males, we asked the males about surgical preferences for a close female relative. It was observed that only 49.60% of the participants believed that a female surgeon is the best for breast cancer surgery, which shows that gender preferences for a surgeon are not a major concern. There have been several studies that explored females' preferences for the gender of their gynecologist-obstetrician [35-37], but only one study has looked into patients' preferences for the gender of their breast surgeons [38]. The findings of the present study demonstrated the same preferences with regard to breast cancer and hemorrhoid surgeries. This suggests that women prefer female surgeons as they believe that they are more gentle/sensitive than male surgeons and merely because they are women. Interestingly, our survey indicated that, while women favored female surgeons, professional and personal qualities were more essential when it came to selecting a choice for a surgeon. We also found that females prefer female surgeons for intimate physical examinations, but when an invasive surgical treatment with a possible health risk is indicated, the surgeon's professional skills are more significant than his/her gender. Moreover, the Saudi population consists of several subpopulations with distinct demographic, religious, and socioeconomic features that may influence the choice of a same-gender healthcare provider. Other research in urban and western societies has indicated that the professionalism of the surgeon, rather than gender, is the most relevant preference factor [22,39]. One of the limitations of responses was unequal gender distribution. However, it is still a significant factor since statistical discrepancies between the genders might lead to ambiguity in the assessment of knowledge, attitude, and practices. Further research is needed to determine the relative importance of these characteristics in influencing the choice of a female surgeon. It was shown that professional qualities, such as surgical ability, experience, and knowledge, were the most critical factors when selecting a surgeon.

## Conclusions

In Saudi Arabia, more than half of the medical graduates are females; however, the percentage of female surgeons is relatively less. We need more female-friendly training programs and better workplace conditions to encourage more women to become surgeons. Government and academic institutions must work together to implement these changes in a multi-pronged fashion. Such an effort could include advocating a reduction in working hours and night shifts, giving monetary assistance for the care of young children and/or creating near-hospital child care facilities, providing acceptable compensation for senior surgeons in public hospitals, and encouraging the participation of female surgeons in medical research and the presence of female physicians in senior clinical and academic posts. Moreover, educational campaigns could make at least some females more receptive to consulting a male surgeon.
